# SIV infection in sooty mangabeys does not impact survival but changes the relative frequency of the main cause of death

**DOI:** 10.1128/mbio.01639-24

**Published:** 2024-09-11

**Authors:** Cristina Ceriani, Brianne Beisner, Maria Crane, Joyce Cohen, Ian N. Moore, Deanna A. Kulpa, Beatrice H. Hahn, Guido Silvestri

**Affiliations:** 1 Emory National Primate Research Center, Emory University, Atlanta, Georgia, USA; 2 Department of Pathology and Laboratory Medicine, Emory School of Medicine, Emory University, Atlanta, Georgia, USA; 3 Departments of Medicine and Microbiology, University of Pennsylvania, Philadelphia, Pennsylvania, USA; University of California, Davis, Davis, California, USA

**Keywords:** SIV, sooty mangabeys, SIV infection, cause of death

## Abstract

Sooty mangabeys (SMs) are natural hosts of simian immunodeficiency virus (SIV) and do not progress to AIDS despite high viral replication. The main factors involved in the benign nature of this infection are (i) low level of immune activation, (ii) relative preservation of specific CD4+ T-cell subsets from direct virus infection, and (iii) absence of microbial translocation from the gut to the systemic circulation. To determine the impact of SIV infection on underlying cause of death, we retrospectively analyzed data from 307 SMs (219 SIV infected and 88 uninfected) housed at the Emory Primate Center that have died between 1986 and 2022. Interestingly, we found that SIV-infected SMs live ~4 years longer than SIV-uninfected SMs, although this result is hard to interpret due to differences in how animals were housed and assigned to specific experimental studies. While the causes of death were not different between SIV-infected and uninfected SMs that died before age 15 (i.e., adult), we found significant differences in the relative frequency of specific causes of death in the elderly population (≥15 years old). Specifically, we observed that SIV-infected SMs were more likely to die from infections but less likely to die from cardiovascular disease (and diabetes in female animals) as compared to uninfected SMs. While confirming the non-pathogenic nature of SIV infection in SMs, these data reveal, for the first time, a qualitative impact of SIV infection on the host physiology that induces a significant change in the mortality pattern in these natural SIV hosts.

**IMPORTANCE:**

In this study, we demonstrate, for the first time, that the natural, non-pathogenic SIV infection of the African monkey SM has a clinical impact which is revealed in terms of main causes of mortality, which are significantly different in the infected animals as compared to the uninfected ones. Indeed, SIV-infected SMs are at higher risk of dying of infectious diseases but appear to be somewhat protected from cardiovascular causes of death. The identification of a specific pattern of mortality associated with the infection suggests that the host-pathogen interaction between SIV and the SM immune system, while non-pathogenic in nature, has a detectable impact on the overall health status of the animals.

## INTRODUCTION

Naturally occurring simian immunodeficiency virus (SIV) infections have been identified in over 40 different African non-human primate species. Interestingly, natural SIV infections of sooty mangabeys (SMs; *Cercocebus atys*) and African green monkeys (*Chlorocebus* spp.) do not lead to progressive CD4+ T-cell loss and development of simian AIDS, even though the virus is equally cytopathic in productively infected CD4+ T cells and plasma viral loads are comparable to those observed during pathogenic HIV infection of humans and SIV infection of rhesus macaques (RMs) (reviewed in references 1–5). In SIV-infected SMs, the resistance to disease progression has been attributed to several non-mutually exclusive specific aspects of the host-pathogen interaction, including (i) the absence of chronic immune activation, (ii) the low levels of microbial translocation from the gut, and (iii) the relative preservation from direct virus infection of specific CD4+ T-cell subsets including central memory cells, stem-cell memory cells, and follicular helper cells (6–15). The fact that natural SIV infection of SMs is non-pathogenic has emerged over many years of clinical and pathological observation of the large colony of infected and uninfected animals housed at the Emory National Primate Research Center (ENPRC), which culminated in a report by Keele et al. indicating that there is no significant difference in the average lifespan of SIV-infected vs uninfected SMs (shown in Fig. S5) in contrast to pathogenic nature of SIVcpz, the immediate precursor of HIV-1, in chimpanzees (16).

Of note, a few reports have also suggested that, with aging, SIV-infected SMs may develop signs of immune decline, including decrease in CD4+ T cells and increased immune activation, which could be partially restored after anti-retroviral treatment, with at least one case of “classic” AIDS reported in a 24-year-old captive SM that was naturally SIV infected (7, 17–20). To better assess the long-term impact of SIV infection on the overall clinical conditions of SMs, we have conducted a systematic analysis of the causes of death in 307 SMs, of which 219 were SIV infected and 88 were uninfected, that were housed at ENPRC and have died of natural causes or elected clinical euthanasia between 1986 and 2022. To the best of our knowledge, this is the first time that such a comprehensive and detailed analysis has been conducted through full and updated access to the ENPRC record of clinical and pathological observations, as well as necropsy reports. While this study clearly confirmed that SIV infection does not reduce the average lifespan of captive SMs, it also revealed that, in elderly animals (defined as older than 15 years), the pattern of causes of death is significantly different between SIV-infected and uninfected animals. In particular, we observed that in SIV, infection is associated with an increased risk of death by infectious diseases and a decreased risk of death by cardiovascular diseases, with no differences between infected and uninfected animals in the risk of death by cancer, diabetes, trauma, and miscellaneous other causes. We believe that the results of this study provide valuable and unprecedented insights into the potential long-term, large-scale impact of SIV infection on the overall health of SMs and, in fact, of any SIV infection in a natural non-human primate host species.

## RESULTS

### Average lifespan of SIV-infected and uninfected SMs

Between January 1986 and December 2022, 307 necropsies were conducted on adult SMs (defined as older than 3 years), who had died either due to natural death or because of euthanasia performed due to the presence of naturally acquired illness. As shown in Table 1, this retrospective analysis included 219 SIV-infected SMs (71.3% of the total), of which 205 were naturally infected (66.8%) and 14 were experimentally infected (4.5%), and 88 were uninfected animals (28.7% of the total). We also found that a total of 116 deaths (37.8% of the total) occurred in adult SMs aged between 3 and 14 years old, including 74 SIV-infected and 42 uninfected animals, while 191 deaths (62.2%) occurred in elderly animals, defined as >15 years old (Fig. S1). As also shown in Table 1, we found a similar distribution of infected and uninfected animals across sex and age groups.

**TABLE 1:** Characteristics of studied sooty mangabeys

Group	Total (%)	SIV+ (%)	SIV− (%)
Overall			
Total	307	219 (71.3)	88 (28.7)
Females	175 (57.0)	126 (41.0)	49 (16.0)
Males	132 (43.0)	93 (30.3)	39 (12.7)
Adult (3–14 years old)			
Total	116 (37.8)	74 (63.8)	42 (36.2)
Females	62 (53.4)	38 (32.8)	24 (20.7)
Males	54 (46.6)	36 (31.0)	18 (15.5)
Elderly (≥15 years old)			
Total	191 (62.2)	145 (75.9)	46 (24.1)
Females	113 (59.2)	88 (46.1)	25 (13.1)
Males	78 (40.8)	57 (29.8)	21 (11.0)

**Fig 1:**
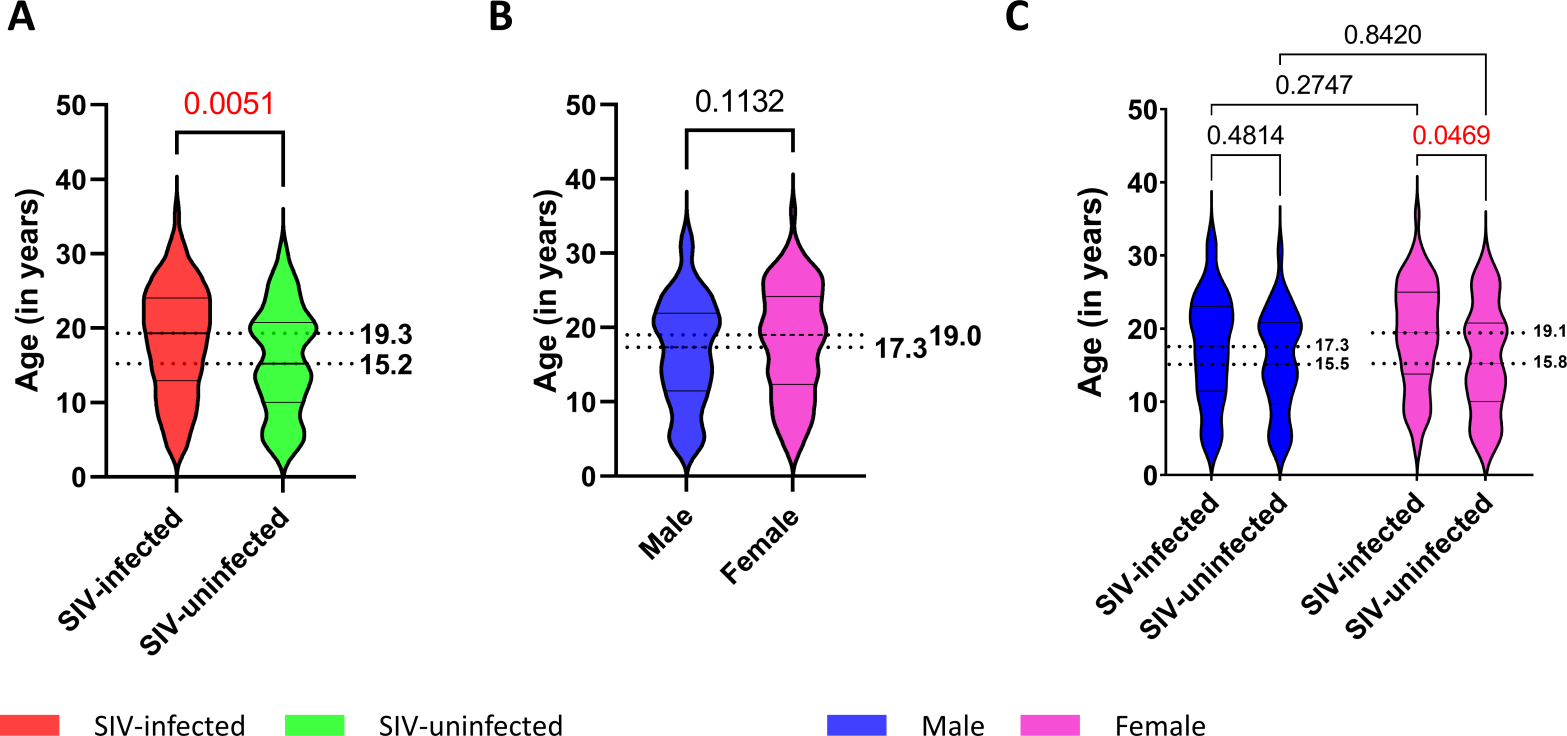
Life expectancy of SMs. (**A**) Age at death in SIV infected (red) and SIV uninfected (green). (**B**) Age at death in males (blue) and females (pink). (**C**) Age at death in male (blue) and female (pink) SIV infected and SIV uninfected. Mann-Whitney test was performed in panels A and B. One-way analysis of variance test was performed in panel C. *P* value indicated and highlighted in red font when <0.05.

We next compared the overall lifespan in SIV-infected vs uninfected SMs and found that, on average, SIV-infected animals lived approximately 4 years longer than SIV-uninfected SMs (median 19.3 and 15.2 years, respectively; *P =* 0.0051; Fig. 1A). As females have a higher predicted survival in several primate species (21) and females have a higher, although not significant, longevity than males in our cohort (median 19.0 and 17.3 years, respectively; *P =* 0.1132; Fig. 1B), we further investigated the impact of SIV infection on life expectancy within sex groups. As shown in Fig. 1C, within the female group, we confirmed a significantly higher life expectancy in SIV-infected animals as compared to uninfected ones (median 19.1 and 15.8 years, respectively; *P =* 0.0469). A similar trend was also observed in male SIV-infected SMs as compared to uninfected males (median 17.3 and 15.5 years, respectively; *P =* 0.4814), although the observed difference did not reach statistical significance. Taken together, these results might suggest that SIV infection could increase the overall life expectancy in SMs. However, it is important to note that the presence of this relatively small difference is hard to interpret because the colonies of SIV-infected and uninfected SMs have been housed separately, undergoing distinct breeding, birth control, and hormone treatment regimens over the course of this 30-year retrospective study. For this reason, we prefer to conclude conservatively that the main message of this data set resides in clearly confirming the non-pathogenic nature of SIV infection of SMs.

### Frequency of causes of death in SMs, all animals, and divided based on age (i.e., adult vs geriatric) and sex

In this study, we defined the cause of death based on the primary pathology diagnosis from the necropsy report of each individual deceased animal as established by the team of veterinarian pathologists at the ENPRC of Emory University. Based on these reports, we have elected to classify causes of death into six major categories: cardiovascular, infectious diseases, neoplasia, diabetes, trauma, and “others/unknown.” While we recognize that there is an unavoidable element of uncertainty in establishing the exact cause of death in the event of complex clinical pictures, we reasoned that by strictly adhering to the definition of cause of death as made by the veterinarian pathologists at the time of necropsy and over a long period of time (1986–2022), we would minimize the risk of biased interpretation of the existing pathology records.

As shown in Table 2, we found that, in terms of relative frequency, the most common cause of death in all SMs (i.e., SIV infected and uninfected) was infectious diseases (24.1%), followed by diabetes (19.5%), neoplasia (12.7%), cardiovascular diseases (9.82%), and trauma (5.2%). In 88 cases (28.7%), the primary cause of death was due to one of a series of relatively rare conditions (i.e., unclassified intestinal diseases, endometriosis, osteoarthritis, and many other conditions, grouped as “others”) or was unable to be determined (i.e., “unknown”), either due to insufficient information in the necropsy report or due to complex clinical pictures in which more than one disease played an important role, thus precluding a unique classification of the causes of death. Of note, throughout the following analyses, others and unknown causes of deaths were grouped together.

**TABLE 2:** Cause of death in the studied sooty mangabeys

Group	Total (%)	Females		Males	
		SIV+ (%)	SIV− (%)	SIV+ (%)	SIV− (%)
Overall					
Total	307	126	49	93	39
Infectious diseases	74 (24.1)	30 (23.8)	10 (20.4)	27 (29.0)	7 (17.9)
Diabetes	60 (19.5)	26 (20.6)	14 (28.6)	17 (18.3)	3 (7.7)
Neoplasia	39 (12.7)	21 (16.7)	5 (10.2)	8 (8.6)	5 (12.8)
Cardiovascular	30 (9.8)	2 (1.6)	5 (10.2)	15 (16.1)	8 (20.5)
Trauma	16 (5.2)	8 (6.3)	3 (6.1)	4 (4.3)	1 (2.6)
Other/unknown	88 (28.7)	39 (31.0)	12 (24.5)	22 (23.7)	15 (38.5)
Adult (3–14 years old)					
Total	116	38	24	36	18
Infectious diseases	48 (41.4)	16 (42.1)	9 (37.5)	16 (44.4)	7 (38.9)
Diabetes	7 (6.0)	0 (0.0)	5 (20.8)	2 (5.6)	0 (0.0)
Neoplasia	9 (7.8)	3 (7.9)	2 (8.3)	2 (5.6)	2 (11.1)
Cardiovascular	3 (2.6)	0 (0.0)	0 (0.0)	2 (5.6)	1 (5.6)
Trauma	10 (8.6)	4 (10.5)	2 (8.3)	4 (11.1)	0 (0.0)
Unknown/other	39 (33.6)	15 (39.5)	6 (25.0)	10 (27.8)	8 (44.4)
Elderly (≥15 years old)					
Total	191	88	25	57	21
Infectious diseases	26 (13.6)	14 (15.9)	1 (4.0)	11 (19.3)	0 (0.0)
Diabetes	54 (28.3)	26 (29.5)	9 (36.0)	15 (26.3)	4 (19.0)
Neoplasia	30 (15.7)	18 (20.5)	3 (12.0)	6 (10.5)	3 (14.3)
Cardiovascular	27 (14.1)	2 (2.3)	5 (20.0)	13 (22.8)	7 (33.3)
Trauma	6 (3.1)	4 (4.5)	1 (4.0)	0 (0.0)	1 (4.8)
Unknown/other	48 (25.1)	24 (27.3)	6 (24.0)	12 (21.1)	6 (28.6)

We next examined the relative frequencies of causes of death in SMs who died as “adult” (defined as between 3 and 14 years of age) or “elderly” (over 15 years of age), and then in female vs male animals. The analysis of these data is shown in Table 2 and reveal, as expected, a significant difference among these two age groups, with a clear increase of mortality caused by cardiovascular diseases and diabetes in the older animals.

### Lower frequency of death by cardiovascular diseases and diabetes, and higher frequency of death by infectious diseases in SIV-infected SMs as compared to uninfected

**Fig 2:**
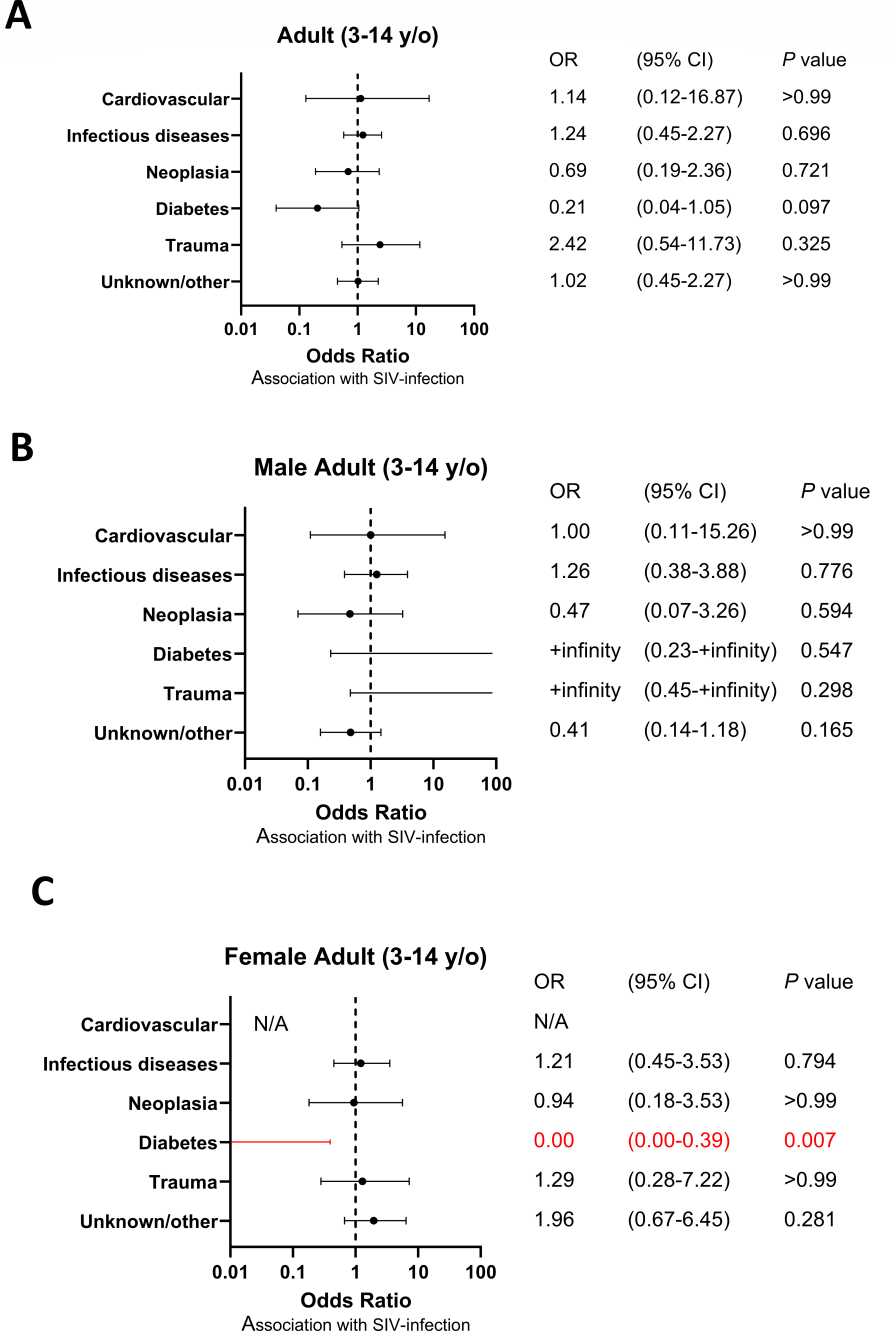
Odds ratio of cause of death in adult (3- to 14-year-old) SIV-infected SMs. (**A**) Odds ratio of causes of death of adult (3- to 14-year-old) SIV-infected SMs not divided by sex, (**B**) in the male group and (**C**) in the female group. Ninety-five percent confidence intervals (CIs) for the odds ratio are shown as error bars on the graph. *P* value less than 0.05 is highlighted in red font.

We next compared the relative frequency of the main categories of causes of death in the SIV-infected SMs (*n* = 219) as compared to the uninfected ones (*n* = 88). We found that, as a whole group, SIV-infected SMs showed a trend toward a lower frequency of death due to cardiovascular disease as compared to uninfected animals (odds ratio [OR] 0.48, 95% confidence interval [CI] 0.23–1.02, *P* = 0.087; Fig. S2A), with this trend reaching statistical significance within the female group (OR 0.14, 95% CI 0.03–0.71, *P* = 0.019; Fig. S2C). We then divided the deaths observed in SIV-infected SMs based on the age of the deceased animals and defined death as “adults,” those occurring between 3 and 14 years of age, and geriatric deaths, those occurring in animals that were 15 years old or older. We first observed that, within the adult group, there was no statistically significant association between SIV infection and any category of cause of death (Fig. 2A). However, we observed, in the same animals, a trend toward decreased risk of death from diabetes (OR 0.21, 95% CI 0.04–1.05, *P =* 0.097; Fig. 2A), which reached statistical significance only in the female group (OR 0.00, 95% CI 0.00–0.39, *P =* 0.007; Fig. 2C).

**Fig 3:**
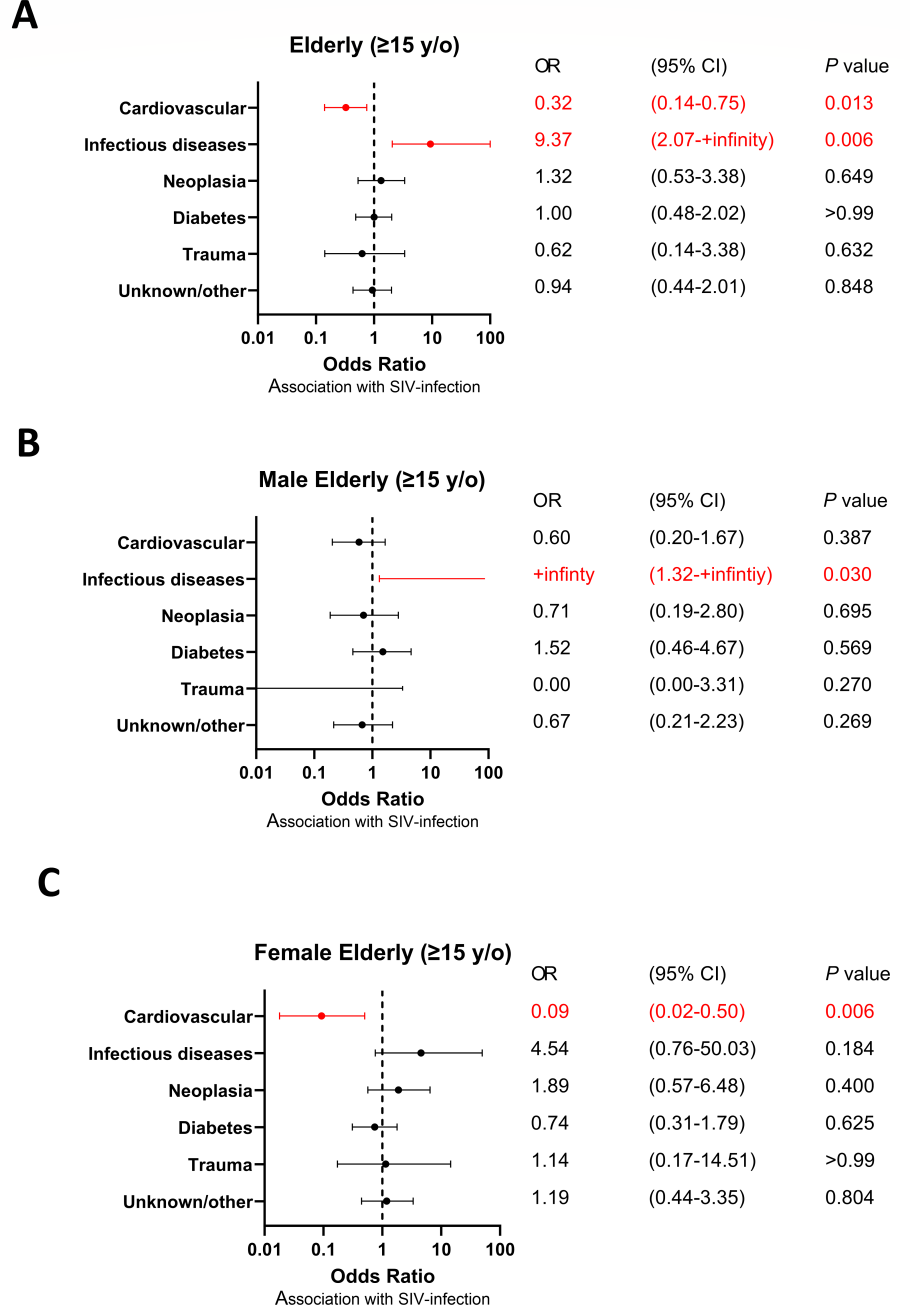
Odds ratio of cause of death in elderly (≥15-year-old) SIV-infected SMs. (**A**) Odds ratio of causes of death in adult (≥15-year-old) SIV-infected SMs not divided by sex, (**B**) in the male group, and (**C**) in the female group. NInety-five percent confidence intervals (CIs) for the odds ratio are shown as error bars on the graph. *P* values less than 0.05 are highlighted in red font.

We next investigated the effect of age of death on the presence of specific changes in the relative frequency of specific causes of death between SIV-infected SMs and uninfected animals. In particular, we focused on the group of SMs that experienced an elderly death (i.e., ≥15 years old). As shown in Fig. 3, we observed that SIV infection was associated with a statistically significant reduction in the frequency of death caused by cardiovascular diseases in both the total group of SMs (OR 0.32, 95% CI 0.14–0.75, *P =* 0.013; Fig. 3A) and when the analysis was conducted within the female animals (OR 0.09, 95% CI 0.02–0.50, *P =* 0.006; Fig. 3C), thus suggesting that sex distribution and female sex’s protective role from cardiovascular disease were not confounding factors for this observation. A similar trend was also observed within the male group but did not reach statistical significance (OR 0.60, 95% CI 0.120–1.67, *P =* 0.387; Fig. 3B).

Interestingly, the comparative analysis of the causes of death between SIV-infected and uninfected SMs as conducted in the group of animals deceased in elderly age also revealed that SIV-infected SMs were significantly more likely to die from infectious diseases (OR +infinity, 95% CI 2.0 to +infinity, *P =* 0.006; Fig. 3A), with the statistical significance confirmed for males (OR +infinity, 95% CI 1.32 to +infinity, *P =* 0.030; Fig. 3B), while a non-significant trend was observed in the female group (OR 4.54, 95% CI 0.76–50.03, *P =* 0.184; Fig. 3C). Table 3 describes in greater detail the deaths caused by infectious diseases in elderly SIV-infected SMs, including, whenever available, the involved pathogen, the time of death, and the tissue and/or organ that was the main site of infection. In this regard, it is important to note that, overall, these analyses do not provide any evidence in favor of the possibility that the increased frequency of death by infectious diseases observed in SIV-infected SMs, which were housed separately from the uninfected animals, was due to specific epidemic events, defined as caused by a common pathogen within a restricted period of time.

**TABLE 3:** Cause of death for infectious diseases in elderly (≥15-year-old) SMs

ID	Sex	SIV	Necropsy data (mo-yr)	Age at death (years)	Pathogen	Type of infection
1	F	+	Sep-92	21		Peritonitis
2	F	+	1999	23	*Escherichia coli*	Septicemia
3	F	+	Jan-00	17		Gastrointestinal
4	M	+	Mar-01	16		Gastrointestinal
5	F	+	Apr-02	16	*Streptococcus pneumoniae*	Septicemia
6	F	+	May-02	17	*Streptococcus pneumoniae*	Meningitis
7	F	+	Aug-02	17	*Mycobacterium fortuitum*	Peritonitis
8	F	+	2002	25	*Streptococcus pneumoniae*	Meningitis
9	F	−	Oct-03	15		Peritonitis
10	F	+	Feb-04	19		Post-trauma
11	F	+	2005	22		Urinary tract
12	M	+	2006	21	*Campylobacter fetus*	Peritonitis
13	M	+	July-09	21		Meningitis
14	M	+	2009	24		Zygomycosis
15	F	+	May-13	24	*Staphylococcus aureus*	Endocarditis
16	M	+	Aug-14	19		Septicemia
17	M	+	Dec-15	24	*Escherichia coli* and *Staphylococcus aureus*	Septicemia
18	M	+	Jan-16	25		Pneumonia
19	F	+	Jan-18	25		Gastrointestinal
20	F	+	Jan-18	36		Pneumonia
21	M	+	June-19	24	*Staphylococcus warneri*	Endocarditis
22	M	+	Nov-19	22	*Staphylococcus warneri*	Endocarditis
23	F	+	Oct-20	26		Pneumonia
24	M	+	Dec-20	24		Pneumonia
25	F	+	Jan-22	29	*Campylobacter fetus*	Gastrointestinal
26	M	+	Aug-22	33		Other

## DISCUSSION

SIV infection in natural hosts, such as SMs, is typically non-pathogenic and does not progress to AIDS. This outcome is in sharp contrast with HIV infection in humans as well as pathogenic SIV_mac_ or SHIV infection of Asian macaques, which represent the most widely used experimental animal model used in studies of HIV/AIDS pathogenesis, prevention, and treatment (1–5). The main factors contributing to the non-pathogenic nature of SIV infection in SMs are the absence of chronic immune activation, the observed low levels of microbial translocation, and a relative preservation of a specific CD4+ T-cell subset from direct virus infection (6–15). While both previous studies (16) and the current comprehensive analysis have clearly demonstrated that, in SMs, SIV infection is not associated with any detectable reduction of the observed lifespan, it is reasonable to posit that some of the observed immunological adaptations to chronic SIV infection have substantially modified the overall “immunological landscape” of the animals. Given the well-established but complex role that the immune system plays in the onset and severity of numerous pathological conditions, we sought to investigate whether SIV infection has a discernible impact on the relative distribution of specific causes of morbidity and mortality in this colony of captive SMs.

In this study, we compared and contrasted the pattern of causes of death among SIV-infected and uninfected SMs and added a sub-analysis of the groups of adults (i.e., ages 3–14) and elderly (i.e., ages 15 and older) deaths, as well as in the groups of female and male animals. This study revealed a clear and previously unrecognized pattern that includes (i) increased frequency of death by infectious diseases in SIV-infected SMs and (ii) increased frequency of death by cardiovascular disease and diabetes in uninfected animals. We wish to emphasize that these observations reached statistical significance despite a relatively small number of events (307 deaths in total), thus indicating a relatively strong biological effect of the studied variable (i.e., SIV infection) on the observed causes of death in SMs. Please also note that we elected not to include in this analysis any pediatric causes deaths (defined as occurring before 3 years of age), as SIV infection of SMs is very rarely transmitted from mother to infant, with the vast majority of naturally SIV-infected SMs becoming seropositive around 2–3 years of age (21, 22).

While the current study is clearly descriptive and observational in nature, with no investigation of specific mechanisms of pathogenesis and their relationship with the immune-pathogenesis of SIV infection, it is tempting to speculate on the reasons why SIV-infected SMs experience a higher frequency of deaths by infectious diseases and a lower frequency of deaths by cardiovascular disease and diabetes. For instance, previous reports suggest a slow decline of CD4+ count with age in SIV-infected SMs compared to uninfected animals (9, 17), which may contribute to the increased frequency of deaths caused by infectious diseases. Similarly, the ability of SIV-infected SMs to rapidly suppress the immune activation caused by the SIV infection (12) may somewhat interfere with the immune responses to specific pathogens and thus predispose elderly animals to succumb to certain infectious diseases. Of note, our study did not reveal any increased frequency of death due to neoplasia in SIV-infected SMs.

In the context of cardiovascular disease, the role of the immune system is well established in humans, and it appears to involve a disrupted balance between pro- and anti-inflammatory mechanisms. In keeping with this notion, chronic immune activation has been linked to onset of cardiovascular diseases in both HIV-infected humans (23–25) and SIV-infected macaques (26). The observed lower frequency of deaths by cardiovascular diseases in SIV-infected SMs emphasizes the striking different outcomes of lentiviral infections between natural and non-natural hosts. Moreover, it is not unreasonable to speculate that the early establishment of an anti-inflammatory and/or immune-regulatory environment in SMs soon after SIV infection might play a role in reducing the overall risk of cardiovascular diseases, which would then translate into a reduced frequency of these diseases as the main cause of death in the SIV-infected animals. The fact that the differences observed between SIV-infected and uninfected SMs reached statistical significance only in the female group may be related to the small sample size of the male SMs populations and/or the possibility of a more pronounced impact of this putative SIV-associated anti-inflammatory effect in female than male SMs. Of note, we also observed a decreased risk of diabetes-related death in the adult female group, and given the well-established link between cardiovascular diseases and diabetes (24), it is possible to hypothesize a common underlying mechanism by which SIV infection is associated with lower frequency of death as caused by these diseases in SIV-infected SMs.

Due to the retrospective study design, samples near time of death were not available, precluding any evaluation of specific comorbidities markers. Future studies in both male and female SMs, with specific focus on inflammatory balance, sex differences, and longitudinal analyses, may help in understanding the mechanism behind the potential protective role of SIV infections against cardiovascular disease and diabetes.

In conclusion, this comprehensive analysis of the causes of death in SMs confirms the non-pathogenic nature of SIV infection in these animals and introduces—for the first time and in a statistically significant way—the concept that the causes of death are different in SIV-infected vs uninfected animals. This observation suggests that specific features of the host-pathogen interaction that emerge during chronic SIV infection may change the overall *in vivo* immunological landscape of SMs with a long-term impact on the risk of developing specific diseases. Additional studies in other SIV natural host species would be valuable to confirm and expand on the findings presented here, while further investigation of the underlying mechanisms may have important implications for our understanding of the immune pathogenesis of HIV infection in humans.

## MATERIALS AND METHODS

### Study population

All animals involved were housed at the ENPRC and maintained in accordance with United States Department of Agriculture and National Institutes of Health guidelines at an Association for Assessment and Accreditation of Laboratory Animal Care accredited facility. The study population consisted of animals living under normal conditions until their natural death or euthanasia based on their clinical status. Necropsies were performed at the ENPRC between January 1986 and December 2022 to reveal the causes of death. The SIV-infected SMs utilized in this study were either naturally infected in the ENPRC colony or experimentally infected before 2007 (*n* = 14). SIV infectious status was determined by SIV PCR of plasma, and negative HIV-2 serology confirmed the absence of SIV infection.

### Pathology

Post-mortem evaluations were performed according to standard necropsy protocol used at the ENPRC between 1986 and 2022. Necropsy involved an external body inspection and evaluation of organ morphology. Organs were evaluated *in situ*, systematically removed, sampled, and placed in 10% neutral buffered formalin for preservation and eventual histological evaluation. Based on anamnestic information, macroscopic and histopathological findings, the final diagnosis of death was determined.

### Statistical analysis

Data were analyzed using GraphPad Prism (version 9.5.1). Mann-Whitney and one-way analysis of variance tests were used as appropriate. The significance level was set at *P* < 0.05.

To evaluate the association between variables, ORs were calculated. The ORs were calculated as the odds of cause of death outcome in SIV-infected SMs divided by the odds of the outcome in the SIV uninfected SMs group. The 95% CI and exact *P* value were calculated.

## SUPPLEMENTAL MATERIAL

Supplemental figures mbio.01639-24-s0001.docx Figures S1 and S2.

ASM does not own the copyrights to Supplemental Material that may be linked to, or accessed through, an article. The authors have granted ASM a non-exclusive, world-wide license to publish the Supplemental Material files. Please contact the corresponding author directly for reuse.
