# Supplementary material for: SIV infection in sooty mangabeys does not impact survival but changes the relative frequency of the main cause of death
Source: mBio. 2024 Sep 11;15(10):e01639-24. doi: 10.1128/mbio.01639-24 (PMC11481876; doi:10.1128/mbio.01639-24)
Supplement: Supplemental figures — Figures S1 and S2. [file mbio.01639-24-s0001.docx]

**Supporting Information for**

**SIV infection in sooty mangabeys does not impact survival but changes the relative frequency of the main cause of death.**

Cristina Ceriani^1^, Brianne Beisner^1^, Maria Crane^1^, Joyce Cohen^1^, Ian N. Moore^1^, Deanna A. Kulpa^1,2^, Beatrice H. Hahn^3^, Guido Silvestri ^1,2,#^.

^1^Emory National Primate Research Center, Emory University, Atlanta, Georgia, USA

^2^Department of Pathology & Laboratory Medicine, Emory School of Medicine, Emory University, Atlanta, Georgia, USA

^3^Departments of Medicine and Microbiology, University of Pennsylvania, Philadelphia, Pennsylvania, USA

^#^Address correspondence to Guido Silvestri, [gsilves@emory.edu](mailto:gsilves@emory.edu)

**This PDF file includes:**

Figure S1 to S2


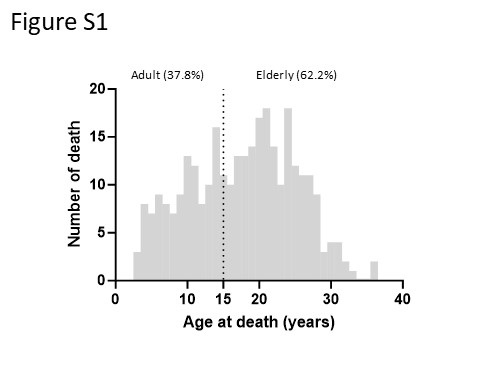


**Figure S1:** SMs distribution of age at time of death.


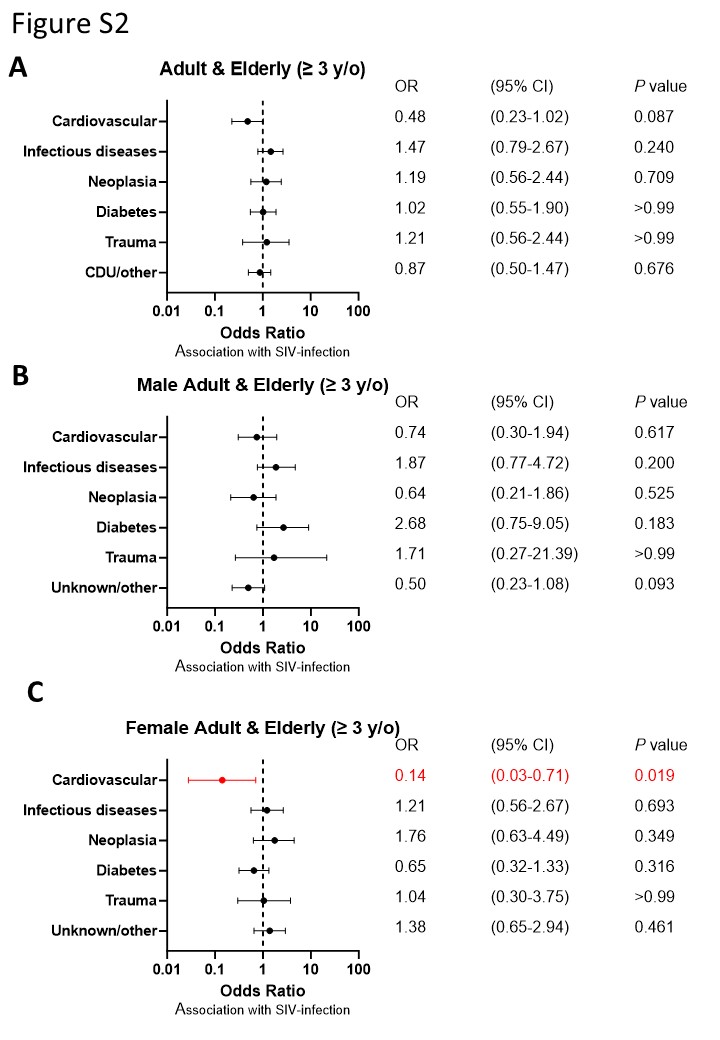


**Figure S2:** Odds ratio of cause of death in all SIV-infected SMs included in the study (≥3 y/o). (A) Odds ratio of causes of death SIV-infected SMs not divided by sex, (B) in the male group and (C) in the female group. Ninety-five percent confidence intervals (CI) for the odds ratio are shown as error bars on the graph and *P* value less than 0.05 is highlighted in red font.
